# BLASTGrabber: a bioinformatic tool for visualization, analysis and sequence selection of massive BLAST data

**DOI:** 10.1186/1471-2105-15-128

**Published:** 2014-05-05

**Authors:** Ralf Stefan Neumann, Surendra Kumar, Thomas Hendricus Augustus Haverkamp, Kamran Shalchian-Tabrizi

**Affiliations:** 1 Section for Genetics and Evolutionary Biology (EVOGENE) and Centre for Epigenetics, Development and Evolution (CEDE), University of Oslo, Oslo, Norway; 2 Current address: Department of Clinical Molecular Biology and Laboratory Science (EpiGen), Division of Medicine, Akershus University Hospital, 1478 Akershus, Norway; 3 Centre of Ecological and Evolutionary synthesis, Department of Biosciences, University of Oslo, Oslo, Norway

**Keywords:** Analysis, BLAST, High-throughput, Taxonomy, Text-mining, Visualization

## Abstract

**Background:**

Advances in sequencing efficiency have vastly increased the sizes of biological sequence databases, including many thousands of genome-sequenced species. The BLAST algorithm remains the main search engine for retrieving sequence information, and must consequently handle data on an unprecedented scale. This has been possible due to high-performance computers and parallel processing. However, the raw BLAST output from contemporary searches involving thousands of queries becomes ill-suited for direct human processing. Few programs attempt to directly visualize and interpret BLAST output; those that do often provide a mere basic structuring of BLAST data.

**Results:**

Here we present a bioinformatics application named BLASTGrabber suitable for high-throughput sequencing analysis. BLASTGrabber, being implemented as a Java application, is OS-independent and includes a user friendly graphical user interface. Text or XML-formatted BLAST output files can be directly imported, displayed and categorized based on BLAST statistics. Query names and FASTA headers can be analysed by text-mining. In addition to visualizing sequence alignments, BLAST data can be ordered as an interactive taxonomy tree. All modes of analysis support selection, export and storage of data. A Java interface-based plugin structure facilitates the addition of customized third party functionality.

**Conclusion:**

The BLASTGrabber application introduces new ways of visualizing and analysing massive BLAST output data by integrating taxonomy identification, text mining capabilities and generic multi-dimensional rendering of BLAST hits. The program aims at a non-expert audience in terms of computer skills; the combination of new functionalities makes the program flexible and useful for a broad range of operations.

## Background

Sequence similarity searches have become an integral part of contemporary life sciences [1]. More than two decades have now passed since the Basic Local Alignment Search Tool (BLAST) was introduced to the bioinformatic community [2,3], constituting a breakthrough for rapid similarity search tools [4]. Despite the staggering changes that have taken place in biology, sequencing and computing technology, BLAST remains the most common used algorithm for sequence similarity searches [5,6]. This is reflected by the exceptionally high numbers of citations for the two original BLAST papers (48632 citations [2], 49238 citations [3]; Google scholar - January 2014). The continued popularity is due to the intuitive appeal of the algorithm [4], its speed and efficiency [7,8], and being supported by a complete, rigorous statistical theory [5].

BLAST has been used for most purposes involving biological sequence searches and alignments, some examples being EST annotation [4,9-11], contig assembly [12,13], genomic fragment reconstruction [14], ORF validation [5], prediction of protein function and origin [4,6], distant homolog [5] and putative ortholog detection [15,16], phylogenetic analysis [8,17,18] and metagenomics [19].

BLAST services can be accessed numerous places on the web and are often free of charge; one of most popular is the BLAST implementation hosted by the National Center for Biotechnology Information (NCBI) [20]. It receives hundreds of thousands requests a day [21,22], and presents BLAST results as textual reports with graphical representations of the calculated alignments.

Such web-based BLAST implementations are convenient to use for the analysis of a small number of sequences. However, both limitations of computational resources and the way results are presented render this approach unfeasible for large BLAST searches that might involve thousands (or even hundreds of thousands) of unique query sequences. In recent years, due to new sequencing technology, high-throughput searches of this magnitude have become a standard situation in many fields of research, such as EST annotation [4], genomics [5], metagenomics [19] and phylogenomics [23]. In addition, projects without high-throughput sequencing data also exhibit a trend towards increasing query numbers. Examples might be all-against-all comparisons useful in EST annotation [4], proteome-against-proteome searches in order to identify orthologs [15], or simply pooling diverse queries together so as to minimize the number of job submissions to an external computing resource.

In order to perform the actual high-throughput BLAST search, it is necessary to use a stand-alone implementation of the BLAST algorithm rather than relying on web-based public installations such as the NCBI resource [5]. Many research institutions have solved this by establishing high-performance computing (HPC) clusters hosting BLAST-related databases and pipelines. Since BLAST scales well when parallelized, such HPC installations can handle large high-throughput sequence input in a reasonable amount of time. The results of high-throughput BLAST runs could still present the user with gigabyte-sized text files, the data volume alone representing a challenge for inexperienced users. For specific research fields, massive BLAST output can be analysed by specialized, user-friendly programs that run on ordinary desktop computers – such as the MEGAN [19] program for metagenomics. For many other research fields however, scientists find suitable tools missing [24]. Surprisingly, this includes generic BLAST output interpreter programs capable of visualizing, analysing and selecting massive BLAST output data.

This reflects a more general problem in the field of biological data visualization and analysis - contemporary biological data generation has outpaced the traditional data processing approaches [20,24]. Some of the important features recently suggested to alleviate the perceived program shortcomings include (amongst others) improved usability, new visual analytical modes, and multi-scale standardized data representations [24]. Especially intriguing is the challenge of visualizing massive amounts of data in a suitable way. New, innovative visual representation techniques are needed that provide both an overview over, and facilitates detailed navigation into, the mass of information in these data sets [20]. Hence, improved visualization technologies are clearly amongst the key aspects of knowledge discovery and data mining [25]; some of which have begun to find their way into mainstream science (for instance, [26-28]). Suitable analysis tools will be in demand as sequencing technology become even more efficient.

We here present a program designed for the effective exploration of BLAST output generated from large scale database searches. It is aimed at an audience of computer non-experts not familiar with programming languages, database retrieval or command-line usage. The program facilitates visualization, analysis and selection of data. Importantly, the application provides new functionalities including taxonomic ordering of data, text search options, multi-dimensional display and a range of possibilities for filtering and downloading of data from public sequence databases. The program, introduction video and additional utilities are freely available for download at http://www.bioportal.no.

## Implementation

**Figure 1:**
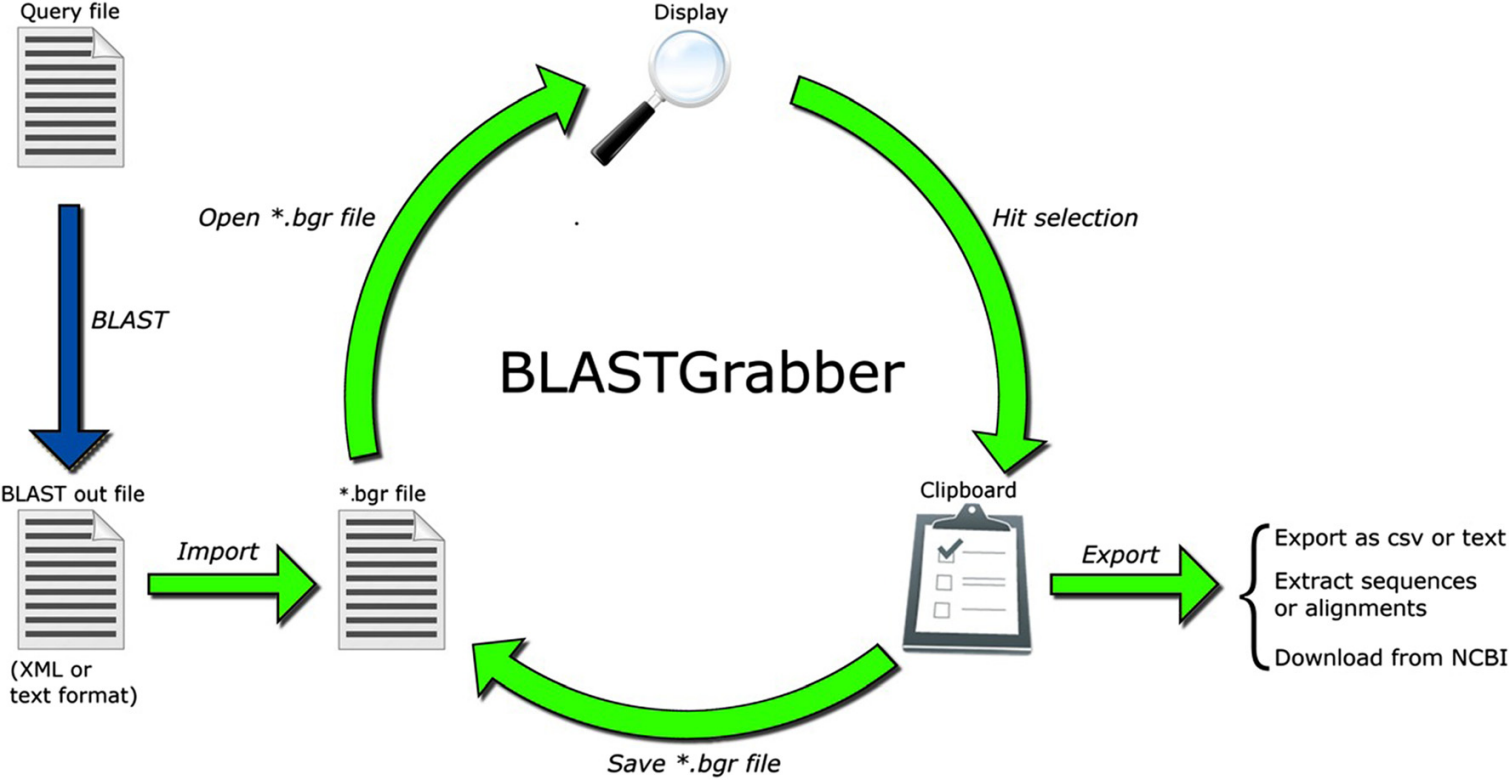
**Workflow and interaction of the BLASTGrabber program.** The text- or XML-formatted BLAST output can be imported directly into BLASTGrabber. BLASTGrabber supports an iterative mode of analysis, with repeated display, selection, saving and re-opening of BLAST data. Sequences of interest can be exported or downloaded.

BLASTGrabber consists of a downloadable desktop Java application capable of visualizing and selecting high-throughput BLAST output. Taxonomic analysis is supported based on mapping of NCBI gi-numbers to taxonomy identifiers, or alternatively parsing the headers of the BLAST hits. Selected BLAST hits and queries can be exported in text format (Figure 1).

### The BLASTGrabber application

The BLASTGrabber desktop application is a GUI-based Java program that will run on Windows-, MacOS- or Linux- based computers. Apart from the Java Runtime Environment (version 1.6 or higher), no other installations are required to run the program. BLASTGrabber will work on low-spec systems including netbooks. For high-throughput data however, 4 GB of memory and a processor comparable to 1.4GHz Core i5 are recommended. BLASTGrabber is installed by extracting the installation zip file containing the BLASTGrabber JAR file and additional files (such as example data sets and NCBI taxonomy file). In order to maximize memory usage, the program can be started from the command line, explicitly specifying the java heap space parameters. Alternatively, double-clicking an included shell file can start BLASTGrabber with 2 GB of maximum heap spaces.

BLASTGrabber uses a custom input file format (*.bgr file format) to represent BLAST output. This file is produced by an import wizard included in the BLASTGrabber application (for details, see User guide in Additional file 1). The wizard will recognize BLAST XML output format, in addition to standard text-formatted BLAST output from both the current BLAST + version and the older BLAST 2.2.* versions. As taxonomy information is not included in the standard BLAST output, taxonomy identifiers will not be included when importing BLAST files.

The *.bgr file is formatted as a text file, and lists the included queries and sequences (names or headers together with internal identifiers). Multiple hit sequences with identical FASTA headers are represented only once. In addition, the file contains BLAST statistics such as e-values and bit scores. NCBI TAXIDs can be appended to the *.bgr file after importing by using the included taxonomy wizard. These allow a downstream taxonomical representation in the BLASTGrabber program.

Upon loading a *.bgr file, the query, sequence and high scoring pair (HSP) attribute objects are instantiated from the *.bgr file and kept in the computer memory until the program is terminated (or another *.bgr file is loaded). Since all the BLAST data is directly available in computer memory, most BLASTGrabber operations complete in a few seconds at the most. Therefore installed memory limits the maximum BLAST data size usable by BLASTGrabber. For BLAST output exceeding these memory limitations, it is possible to break apart the offending BLAST file into multiple smaller parts. These can be imported and opened individually, and possibly merged together again at a later point in the analysis. The effectual maximum BLAST output size can be doubled using this technique.

The BLASTGrabber installation folder includes the NCBI taxonomy structure compressed into a custom binary format. In order to keep the start-up time and memory overhead as low as possible, only scientific taxonomy names are included. Loading the taxonomy happens automatically at BLASTGrabber start-up, but can be cancelled by the user in order to preserve memory or improve BLASTGrabber start-up time. The BLASTGrabber taxonomy file can be updated by downloading the NCBI taxonomy files and using the included update functionality.

BLASTGrabber includes text search options; these are implemented using the Java regular expression (regex) API. Graphical representations of hit alignments are based on HSP attributes describing the start and end of the BLAST-generated alignments. No mismatches or gaps are indicated since this information is not included in the *.bgr file. However, sequences and alignments for selected hits can be downloaded from NCBI or extracted from the original BLAST output and query files.

BLASTGrabber architecture supports third party plugin development not depending on BLASTGrabber source code. Communication to and from the plugin is defined by two specific interfaces; JAR folders in the “plugins” subfolder featuring these required interfaces are loaded and activated at BLASTGrabber start-up (API description and plugin example are provided at the BLASTGrabber download site).

### Example datasets

Two queries representing the Fibronectin-3 (Pfam id: PF00041) and I-set (Pfam id: PF07679) domains on the *Drosophila melanogaster* “Down syndrome cell adhesion molecule” protein (uniprot id: A1Z6X1_DROME) were used in a BLAST search against the *Drosophila* uniprot reference proteome. These two motifs are found multiple times next to each other in various *Drosophila* multi-domain proteins and are well suited to illustrated sequence alignment visualization in BLASTGrabber.

The BLASTGrabber visualization and analysis of the example datasets was compared to the JAMBLAST [29] and BlastViewer (Korilog SARL, Questembert, France) programs. As JAMBLAST utilizes a customary csv format (*.bls) produced by the accompanying NOBLAST [29] BLAST implementation, identical searches were performed producing the required BLAST output formats. In addition to the NOBLAST *.bls format required by JAMBLAST, BLAST XML output is required for BlastViewer.

In order demonstrate taxonomy display, a BLAST search (using the BLOSUM45 matrix; expect threshold at e-value = 1) was performed against the NCBI “nr” database using the two “Fibronectin-3” and “I-set” queries mentioned above.

## Results and discussion

### User-friendly visualization and analysis

Despite the continuing high popularity of the BLAST algorithm and ever-increasing data loads, there is a perceived lack of high-throughput-capable, user-friendly generic BLAST output visualisation tools. BLASTGrabber is designed to be usable by computer non-experts and implements a truly multi-query mode-of-analysis which is backed by visual displays. The included taxonomy wizard adds taxonomy identifiers to the BLAST data, allowing the downstream integration of sequence descriptions, origins and hit quality assessment. Also, the BLASTGrabber architecture facilitates the development of third-party plugins, opening new possibilities for BLAST-related analysis algorithms. Some aspects of BLASTGrabber overlap with the JAMBLAST and BlastViewer programs functionalities. Just as BLASTGrabber, both are free and user-friendly Java programs that visualize BLAST files. While supporting many additional analysis features when compared to existing similar programs, we find BLASTGrabber to perform as well or better in terms of data input capacity and speed, all the time retaining minimal installation requirements and an intuitive and user-friendly interface.

### BLASTGrabber uses a matrix viewer of hits and queries

**Figure 2:**
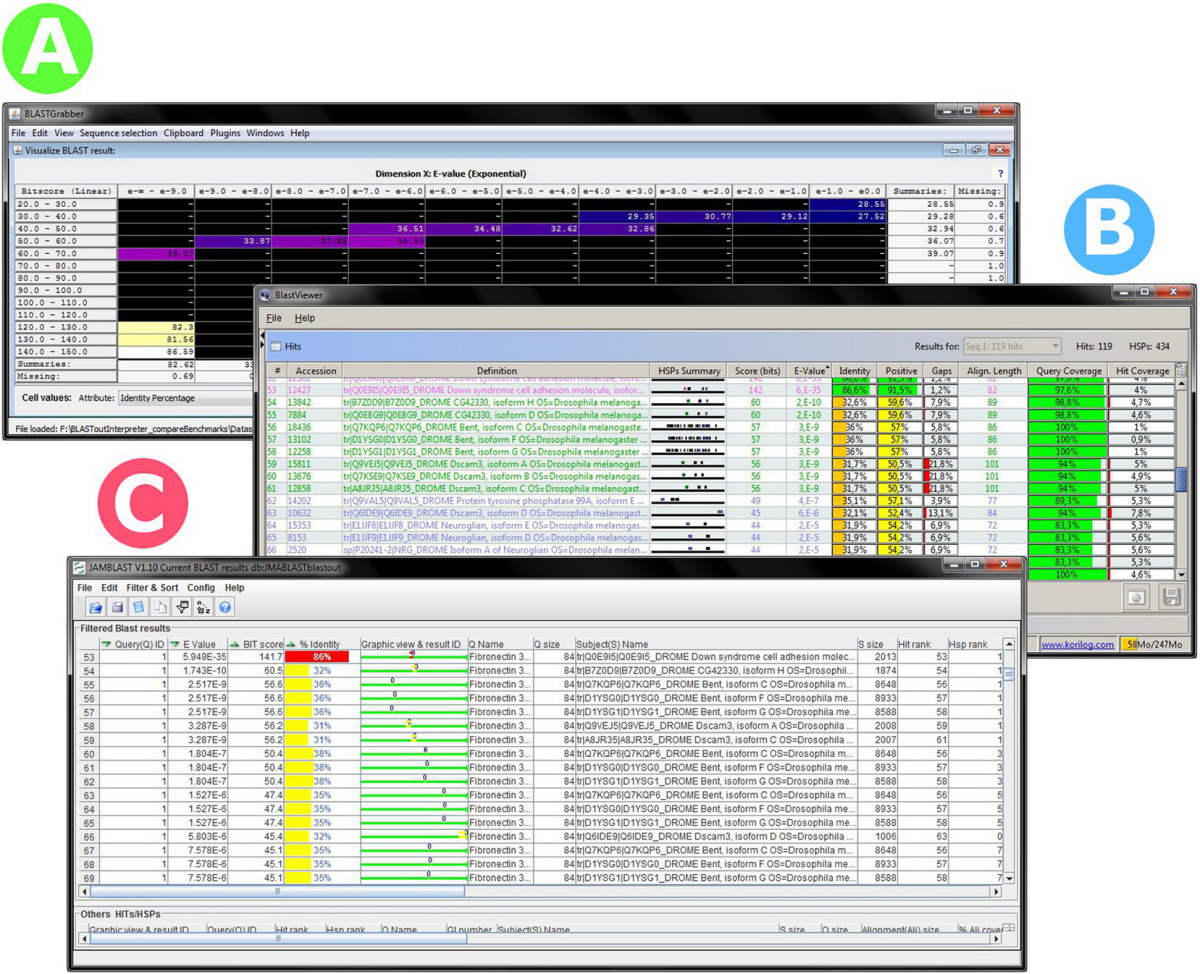
**The BLASTGrabber, BlastViewer and JAMBLAST program ordering BLAST hits according to high-scoring hit attributes. A)** Hits are sorted according to e-values and bit scores along the horizontal and vertical axes in the BLASTGrabber matrix viewer. The average identity percentages are displayed inside the table cells. Heat map cell rendering has been applied, reflecting the identity percentage magnitudes. **B)** In the BlastViewer program, the hits for the selected query (“Seq 1”) are sorted by clicking on the corresponding column headers. **C)** The JAMBLAST program supports explicit multiple sorting, as indicated by the arrows shown in the corresponding column headers. Note the additional sorting on the query id column header necessary due to JAMBLAST displaying both query and hit information on the same table row.

BLAST-related programs often display hits and queries as a hierarchical structure, where each query is associated with an ordered list of hits for that query. Each hit in turn can possibly contain multiple HSPs for that hit and query; often graphical representations of the hit alignments are displayed. Ordering of hit and HSP lists is done by some measurement of similarity (often configurable), such as e-values or bit scores. This analytical structure, which demands the selection of individual queries to inspect associated hits, is so pervasive it has been claimed to be almost without alternatives [20].The visualisation structure adopted in BLASTGrabber differs fundamentally from this hierarchical approach. Using the matrix viewer (Figure 2A), hits are distributed in table rows and columns depending on two selected HSP attributes (in this example, two measures of similarity such as e-values, bit scores etc.). The cell content thus represents all hits (i.e. queries with their associated sequences) that conform to the cell specifics (for instance, e-values between e-5 and e-10 and bit scores between 200 and 250). A third attribute statistic is displayed in the cells themselves, the default being the number of hits therein, but other statistics such as average identities are available. Heat map rendering based on these values can be applied. Row and column sort in response to header clicks; lexicographical sorting is implicitly possible by selecting the desired attributes for row and column intervals, in addition to cell statistics.

This hit-focused (in contrast to query-focused) display mode allows the inspection of hundreds of thousands of hits at a glance, distributed across a few table cells only. Cell contents (i.e. the queries and hits) are easily selected, either by manually clicking on cells of interest, or else defining cut-off values for cell statistics (for instance selecting all hit with e-values below e-5). Selected cells are “grabbed”, i.e. copied to the BLASTGrabber clipboards. Clipboard contents are persistable as *.bgr files, allowing iterative purifying selection by *.bgr file opening, subset selection and persistence.In contrast to BLASTGrabber, the BlastViewer program displays hits and queries as a hierarchical structure. When using multiple-query BLAST output each query must be selected before the associated hits are displayed (Figure 2B). The hit list is sortable by any HSP attribute by simply clicking a column header; top-scoring hits can be exported to a text editor in csv format. Lexicographical sorting (for instance based on e-values, bit scores and identity percentages in this priority) can be accomplished by clicking column headers in the reverse order of the desired priority. BlastViewer visualizes all the BLAST attributes for each of the hits, but because queries have to be selected one at the time without any filtering options, it becomes virtually impossible to use for analysis of thousands, or even hundreds of thousand, queries.JAMBLAST also employs a hierarchical structure but has a slightly different visualization mode than BlastViewer, displaying all queries and top scoring hits in the same table (Figure 2C). The program supports lexicographical ordering by the explicit selection of the HSP attributes sort order. The best hits for multiple queries can be selected in one go with subsequent export to a text editor program. Without clustering multiple hits into fewer categories (such as dividing hits into significant and insignificant e-value categories), it is virtually impossible to extract relevant information from high-throughput data.

### Alignment visualization

Most BLAST output interpreter programs provide a graphical representation of the BLAST-generated alignment, i.e. the sequence overlap between query and hit. In addition to displaying hits as they are aligned against a selected query, BLASTGrabber can also do the opposite; i.e. display multiple queries overlapping one selected hit. Thus, for instance the position of multiple genes (queries) along one chromosome (one hit) can be displayed.

**Figure 3:**
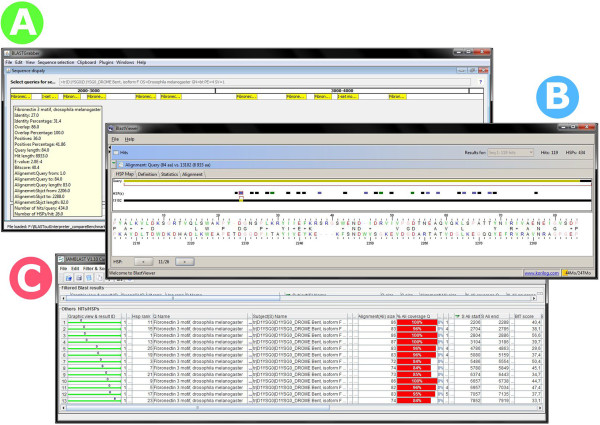
**Comparing the graphical representations of BLAST alignment by the BLASTGrabber, BlastViewer and JAMBLAST programs. A)** HSP alignments from both “Fibronectin-3 motif” and “I-Set motif” queries aligned against the “D1YSG0_DROME Bent” protein sequence in the BLASTGrabber program. Triggered by positioning the mouse over it, details for the first HSP are displayed. Based on a selected HSP attribute such as e-values or bit scores, the HSP representations can be colour-coded as a heat map (not shown because this would hide the HSP query names) **B)** HSP for the “Fibronectin-3 motif” query aligned against same protein in the BlastViewer program. In this program, HSPs for multiple queries cannot be displayed in the same graphical representation. The HSP overlaps are colour-coded (purple, green and black) so as to reflect significance categories. **C)** The same HSPs (“Fibronectin-3 motif” vs. “D1YSG0_DROME Bent”) as displayed by the JAMBLAST program. Again, alignments from multiple queries cannot be displayed along the same sequence.

**Figure 4:**
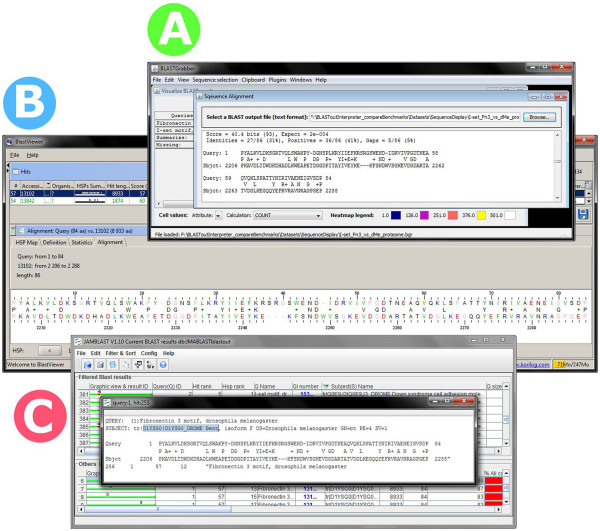
**BLAST alignment sequences as presented by the three BLAST interpreter programs. A)** Alignment details for a selected hit can be extracted from the original BLAST output file in BLASTGrabber. **B)** After selecting the hit for a given query, alignment details for the HSPs are given in the lower half of the BlastViewer screen. The alignment details cannot, however, be copied or exported. **C)** Likewise, alignment details for a select hit can be visualized in the JAMBLAST program if the actual alignments have been included in the custom NOBLAST output.

“Grabbing” is integrated across BLASTGrabber viewers; hits represented by the displayed alignments can be copied to the clipboards just as for the matrix viewer. Both BLASTGrabber and BlastViewer support a colour-coding schema reflecting the statistical significance of the hit (in BLASTGrabber this is part of a generic heat map rendering based on any selected HSP attribute).BlastViewer (Figure 3B) restricts alignment visualization to one query/sequences pair, displaying all HSPs as coloured bars along the sequence representation. JAMBLAST (Figure 3C) includes just one such alignment at the time; multiple hits for one query cannot be included in one graphical representation. Neither of these two programs can display multiple queries for one selected hit (an included option in BLASTGrabber).All three programs can include the display of alignments in text format (see Figure 4). In BLASTGrabber the alignment is extracted from the original BLAST output file; the JAMBLAST database includes alignments only if the relevant option was included when running NOBLAST. The BlastViewer alignment is read-only and cannot be copied.

### Text searches with the description viewer

**Figure 5:**
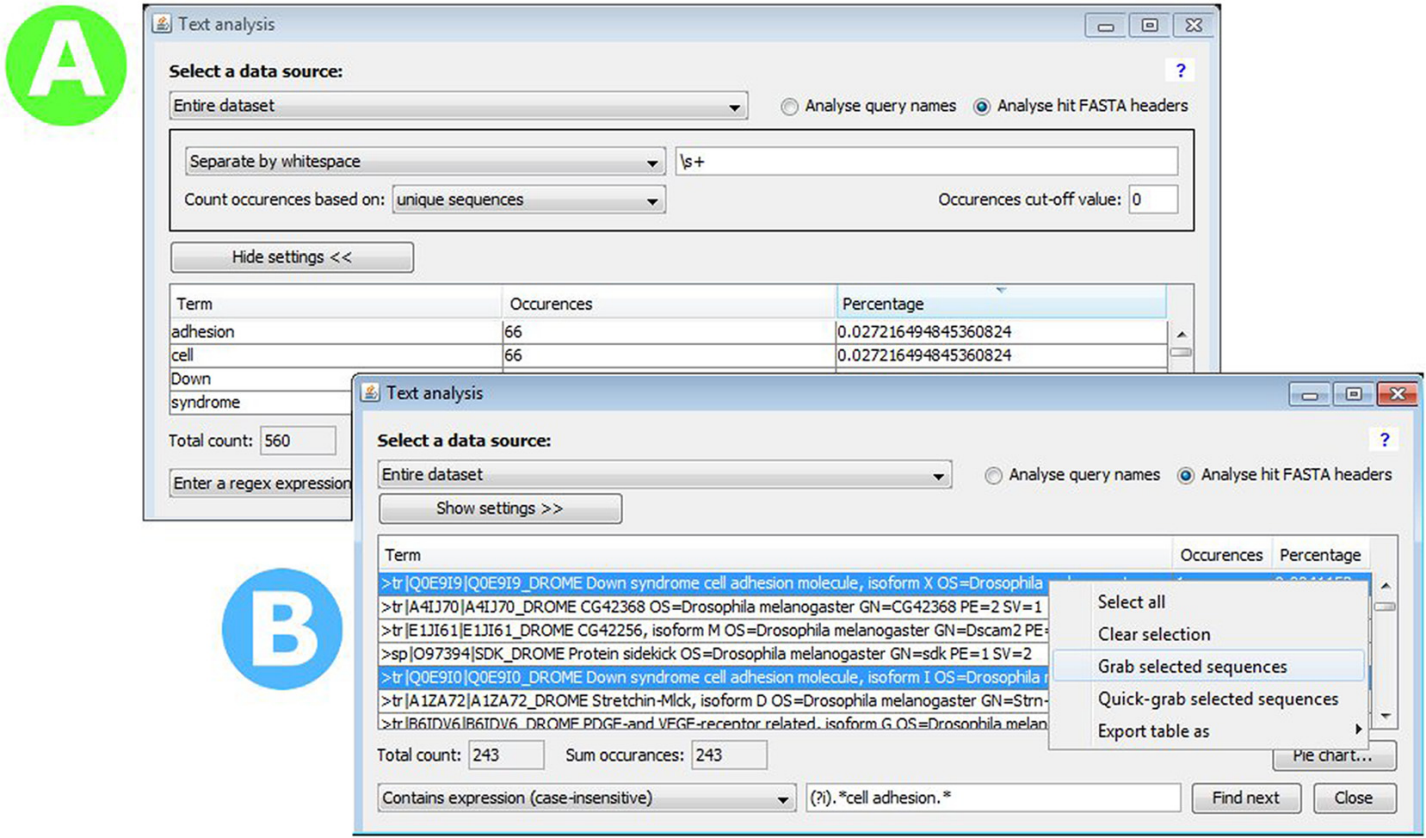
**The BLASTGrabber description viewer. A)** The descriptions in the sequence headers are broken apart by whitespaces, allowing a per-word analysis of the BLAST results. Many hits clearly belong to the “Cell adhesion” and “Down syndrome” protein categories. **B)** Unique headers are displayed in the description viewer. Using a regular expression search term (user-friendly regex templates are provided), all headers containing the “cell adhesion” term are selected, and can now be copied to the BLASTGrabber clipboard.

JAMBLAST and BlastViewer, like most BLAST output interpreter programs, do not support text searches in the sequence FASTA headers. The BLASTGrabber description viewer (Figure 5) however, supports flexible text search with subsequent hit selection in both headers and BLAST query names. Using regex search terms, matching entries can be selected and copied to BLASTGrabber clipboards. Suitable regex templates are provided, demanding no prior knowledge of Java regex syntax.

In addition to performing searches in the complete headers or query names, these can be split into individual words. This allows determination of the relative frequencies of keywords of interest, such as enzyme or species names. Word splitting is regex-based and fully configurable.

### Taxonomical ordering of data in the matrix viewer

**Figure 6:**
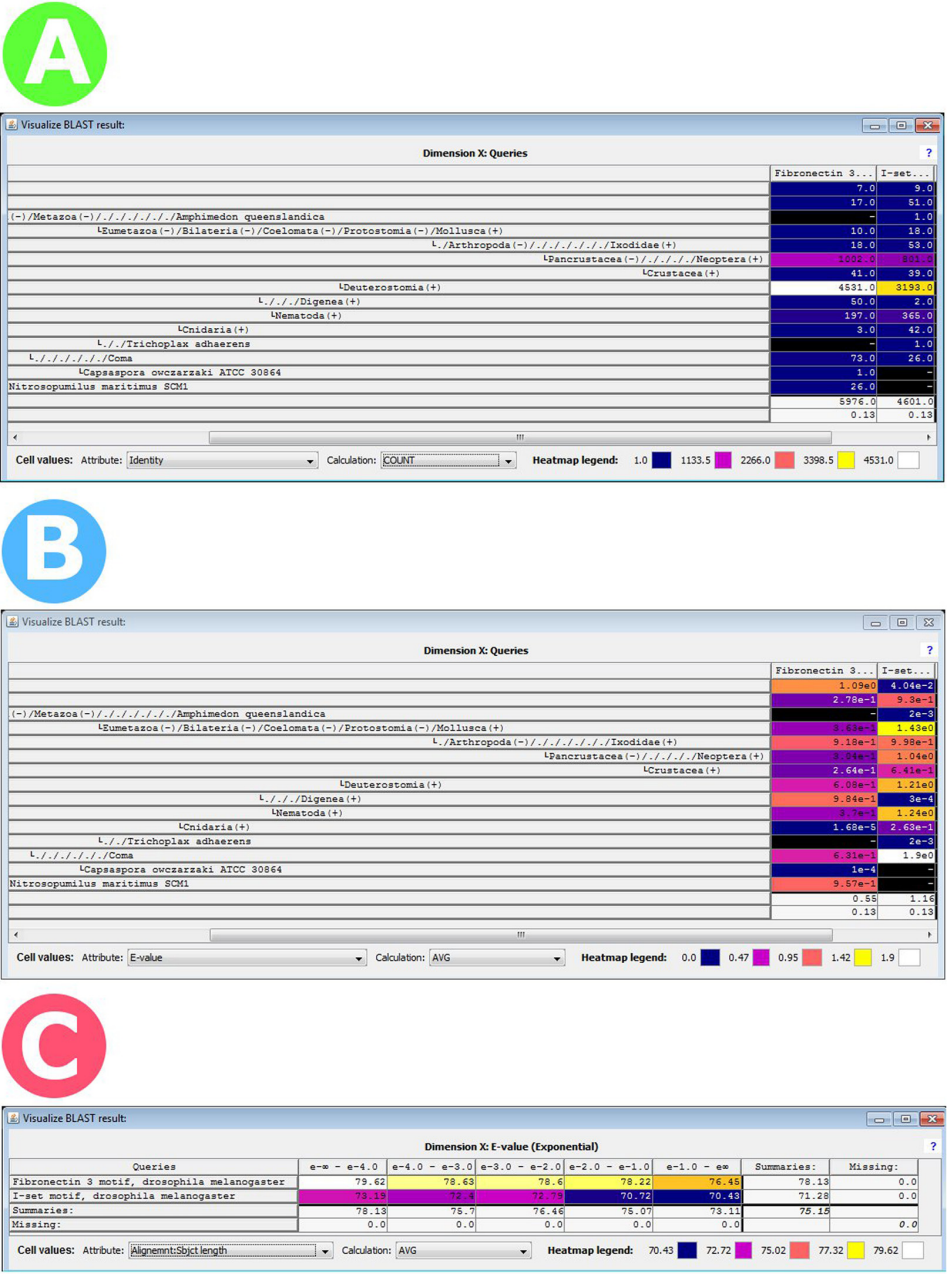
**The NCBI taxonomy as displayed by the BLASTGrabber matrix viewer. A)** The taxonomic distribution for the two Fibronectin-3 and I-Set motifs as queried against the NCBI nr database, represented by the absolute number of hits per query/taxonomic unit. Heat map display mode has been chosen. Most taxonomic groups (such as “Deuterostomia” or “Nematoda”) are collapsed, as indicated by the “+” (expandable) sign. The “Amphimedon Queenslandica”, “Trichoplax adhaerens” and “Capsaspora owczarzaki” species are fully expanded. By far the most hits are found inside the “Deuterostomia” taxonomic group. **B)** Average e-values are displayed inside the same taxonomic setup. Many groups are characterized by rather dubious average e-values, such as the “Deuterostomia”-specific hits (6.08e-1 and 1.21e0 for the two queries). **C)** Analysis of the distribution of e-values for the two queries. Numbers of hits are shown for five e-value intervals. It is now possible to select the leftmost column (containing significant hits with e-values better than e-4), saving and re-loading the selection using the clipboard functionality, so as to recreate the above taxonomic setup with significant hits only.

All biological sequences reflect evolutionary events such as adaption, speciation or separation. Thus they are best classified according to a taxonomical order believed to fit these events. The classification of organisms has been revolutionized by DNA sequence analysis, which has become one of the primary means of taxonomic identification of species [30]. Besides the obvious goal of constructing phylogenies and an evolutionary tree of life, taxonomical sequence information can be utilized in many diverse tasks. These include ecological studies and metagenomics of un-culturable micro-organisms [31], the removal of contamination (for instance sequences with bacterial origin) from shotgun or contig datasets, or the study of horizontal gene transfer. Taxonomy even facilitates tasks such as protein domain discovery [32] or protein fold recognition [33].In BLASTGrabber, the NCBI taxonomy is fully integrated into the matrix viewer (Figure 6), displayed as an interactive tree structure. Along the matrix viewer vertical axis, taxonomy nodes can be expanded or collapsed at will, facilitating selection or taxonomic annotation at a level higher than the species level. The horizontal axis can harbour intervals for any BLAST HSP attribute, or even the queries or hit sequences themselves. Taxonomic distributions can easily be assessed by ordering the columns according to frequency counts or accumulative distributions, possibly using the heat map display mode. Hit selection and “grabbing” is fully supported. For instance hits identified as cnidarian sequences containing the “Fibronectin 3” motif can be “grabbed” by selecting the corresponding cell. Alternatively, undesired groups such as for instance “nematode” can be excluded from further analysis (Figure 6A and B). Neither BlastViewer nor JAMBLAST support this mode of analysis.

### Splitting and merging files

Contemporary high-throughput projects may generate millions of sequences used as queries in BLAST similarity searches (for one example, see [13]). Depending on the installed computer memory, BLASTGrabber can handle BLAST output containing a few hundred thousand queries. If the relevant data set is too substantial to be imported into BLASTGrabber, the BLAST file can be split into smaller parts; each single part can then be imported individually into BLASTGrabber. Subsequently, several *.bgr files can be merged to join previously disjoint data sets.

### BLASTGrabber performance

Given ever-increasing BLAST output sizes, the ability to handle such data on a normal desktop computer will be considered crucial by many users. In implementing BLASTGrabber, care was taken to minimize the data representation memory footprint. The validity of the approach was confirmed when comparing BLASTGrabber to the BlastViewer and JAMBLAST applications (Table 1). Compared to BlastViewer and JAMBLAST, maximum BLASTGrabber capacity was about twice as high.

**Table 1:** Maximum data capacities of the BLAST interpreter programs*

**Program**	**Maximal capacity**	**Input file**
**BlastViewer**	~1.200.000 sequences	XML file (BLAST XML format, size ~2.1 GB )
**JAMBLAST**	~1.000.000 sequences	bls file (NOBLAST csv format, size ~290 MB)
**BLASTGrabber**	~2.200.000 sequences	bgr file (custom text format, size ~ 280 MB)

*as tested on a laptop computer; 1.4 GHz Core i5 2537 M processor, 4 GB of memory, SSD hard drive. Java heap space was set to 4 GB. On average, BLAST data consisted of 100 sequences pr. query.

Once BLAST data has been loaded, all three programs were quite responsive and performed most operations within seconds, even when being used close to their maximum capacities (for performance examples of typical BLASTGrabber operations, see Table 2). Data loading by itself also executed quite rapidly as long as the input file sizes did not exceed ~75 percent of the maximal capacity.

**Table 2:** Performance examples of typical BLASTGrabber operations*

**BLAST file**	~90.000 queries (file size 640 MB )
Import (creation of *.bgr file)	~5 minutes
Resulting *.bgr file	~45.000 queries** (file size 140 MB).
Opening the *.bgr file	~30 seconds
**Opening matrix viewer, 10 rows × 10 cols (100 cells)**	~1 second
**Opening matrix viewer, all queries × 10 cols (450.000 cells)**	~2 seconds
**Selecting a significant subset (e-value < e-5)**	<1 second
**Saving the selected significant subset**	~10 seconds

*as tested on a laptop computer; 1.4 GHz Core i5 2537 M processor, 4 GB of memory, SSD hard drive.

**on average 23 hits pr. query; queries without hits were excluded from the BLASTGrabber input file.

## Conclusions

Along with the rest of bioinformatics, performing BLAST searches has changed substantially over the past two decades. The default textual BLAST output is well adapted to single-query BLAST runs, but next-generation sequencing technologies and growing database sizes demand advanced visualization and analysis capabilities. At the same time, an increasing number of biologists without expert knowledge of programming languages or database retrieval techniques need to work with BLAST and BLAST-related tools.

We believe that the BLASTGrabber program can serve as such a tool. It enables the handling of large amounts of data on desktop computers, while at the same time delivering new and advanced visualization styles. In addition, the flexible text-mining options provide easy selection of BLAST hits or queries. Finally, the integrated taxonomy display can add an extra dimension to traditional sequence similarity searches.

## Availability and requirements

**Project name:** BLASTGrabber.

**Project home page:**http://www.bioportal.no.

This web address also hosts the BLASTGrabber user guide and a tutorial video demonstrating the use of BLASTGrabber. The BLASTGrabber zip file contains both sample files and the user guide.

**Operating system:** Platform independent.

**Programming language:** Java.

**Other requirements:** Java Runtime Environment (version 1.6 or higher).

**License:** Creative Commons Attribution-NonCommercial-ShareAlike 3.0 Unported License.

**Any restrictions to use by non-academics:** None.

## Abbreviations

BLAST: Basic Local Alignment Search Tool; Csv: Comma-separated values; GB: Giga-bytes; JAR: Java Archive; HPC: High-performance computing; HSP: High-scoring pair; MB: Mega-bytes; NCBI: National Center for Biotechnology Information; Regex: Regular expressions; UoO: University of Oslo.

## Competing interests

The authors declare that they have no competing interests.

## Authors’ contributions

Concept and design: RSN, SK, KST. Programming: RSN, SK. Testing: RSN, SK, THAH, KST. Drafting manuscript: RSN, SK. Critical revisions: RSN, SK, THAH, KST. Final manuscript approval: RSN, SK, THAH, KST.

## Supplementary Material

Additional file 1 BLASTGrabber User Guide. Click here for file
