# Supplementary material for: BLASTGrabber: a bioinformatic tool for visualization, analysis and sequence selection of massive BLAST data
Source: BMC Bioinformatics. 2014 May 5;15:128. doi: 10.1186/1471-2105-15-128 (PMC4062517; doi:10.1186/1471-2105-15-128)
Supplement: Additional file 1 — BLASTGrabber User Guide. [file 1471-2105-15-128-S1.pdf]

BLASTGrabber User Guide

By Ralf Neumann and Surendra Kumar

Content

Installing and starting the BLASTGrabber application ... 2

- Installation ... 2
- Running BLASTGrabber ... 2

General BLASTGrabber functionality ... 3

- The mapping of HSP attributes: ... 3
- Standard functionality ... 3

Importing your BLAST output file ... 4

Advanced import functionality ... 6

- Splitting a large BLAST output file ... 6
- Merging several BLASTGrabber input files ... 7
- Inferring taxonomy identifiers ... 8

A first look at your data ... 9

- Summary ... 9
- Taxonomy ... 10

Analysing your data in the sequence viewer ... 11

Analysing your data in the matrix viewer ... 14

- Setting up the matrix ... 14
- Matrix visualization and data selection ... 16

Analysing sequence descriptions with the description viewer ... 17

- Using the description viewer ... 17
- Configuring the description viewer settings ... 18

Working with clipboards ... 19

- Displaying selected sequences ... 19
- Clipboard administration ... 21

Editing your preferences ... 22

Appendix ... 23

- BLASTGrabber plugin architecture ... 23

Installing and starting the BLASTGrabber application

Installation

Requirements: In order to run the BLASTGrabber program, you need Java 1.6 (or later) installed on your computer. BLASTGrabber is designed to run on Windows, Mac OS and Linux computers.

Installation: The Java BLASTGrabber application is distributed as a zipped folder containing the "BLASTGrabber.jar" program file and additional files. Installation consists simply of extracting this folder to a suitable location (for instance, "c:\Program files\BLASTGrabber").

Verify your installation: Double-click on the "BLASTGrabber.jar" file located in the extracted BLASTGrabber folder. Once the start-up window is displayed, click the "Skip loading taxonomy"-button. This will close the start-up window and display the BLASTGrabber user interface.

Running BLASTGrabber

Double-clicking the "BLASTGrabber.jar" file as described above will start the program using the Java default memory settings. On most systems, default memory allocation is too low to allow productive use of BLASTGrabber (hence the instructions to skip the loading of taxonomy information). BLASTGrabber can also be started by executing the included "BLASTGrabber.bat" (for Windows) or "BLASTGrabber" (for MacOS) files. This allocates up to 2 GB of memory to BLASTGrabber.

Alternatively, BLASTGrabber can be started from the terminal window (the "Command" window on windows systems) and, after navigating to the extracted BLASTGrabber folder, typing

```
java -Xms512m -Xmx512m -jar BLASTGrabber.jar
```

at the command prompt. This will start the program with a 512 megabyte memory allocation. You can increase the numbers if your system supports higher memory (in theory, you should be able to allocate as much memory as you have installed on your system, but probably you will have to use somewhat lower numbers. An error message will be given if the requested memory allocation exceeds system constraints).

As described above, a start-up window will be displayed upon starting the program, and it remains visible while taxonomy information is loaded. You can choose to skip loading the taxonomy (by pressing the button or closing the window). Doing this will start the program more rapidly, and also lower memory consumption. If your BLAST output file contains BLASTGrabber compatible taxonomy information, however, you might want to wait until the taxonomy has been loaded, so as to be able to use that information in you subsequent analyses.

General BLASTGrabber functionality

The mapping of HSP attributes:

The BLASTGrabber HSP attributes associated with a BLAST hit are all derived from the BLAST output file used to produce the BLASTGrabber input file. They can be either directly imported or else derived from a combination of imported values. The following image shows an example of the mapping between a BLAST output file and BLASTGrabber HSP attributes:

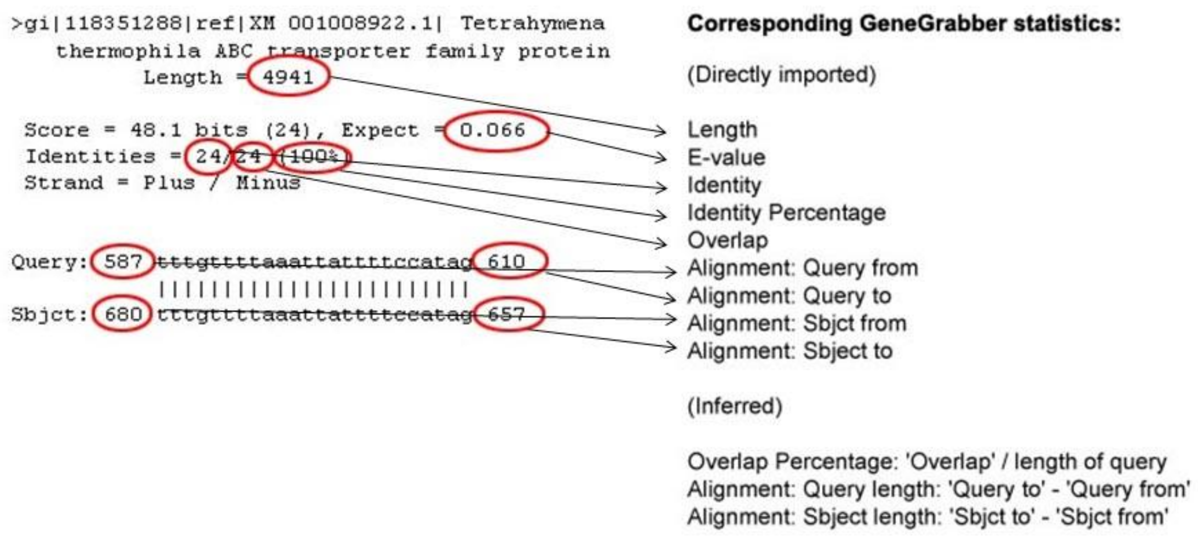

(The specific BLASTGrabber HSP attributes used might vary somewhat, based upon the type of BLAST output file imported, BLASTGrabber version and mode of import. For instance, a BLAST output file produced with the `blastp` program contains the ‘Positives’ attributes which is not available for nucleotide BLAST programs.)

Standard functionality

BLASTGrabber supports most of the standard editing hotkey combinations, such as the ‘Ctrl+A’ combination (select all), ‘Ctrl+C’ (copy) or ‘Ctrl+V’ (paste - Window users), both in text boxes and tables. Arrow keys ‘right’ and ‘left’can be used to navigate in tables and expand or collapse rows, and most tables can be sorted by clicking on column headers.

Importing your BLAST output file

Before you can visualize your BLAST output data in BLASTGrabber, you must create an input file that BLASTGrabber can understand. You do this by selecting '**File->Import file...**' which opens the import window:

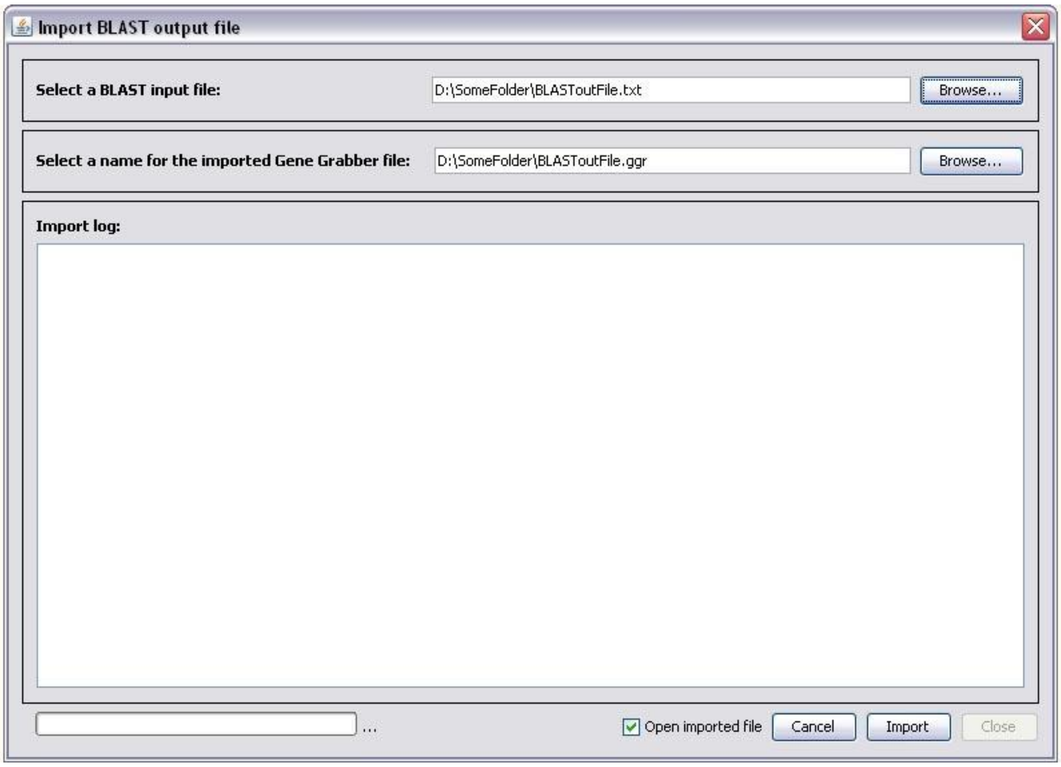

Here, you enter the path and filename of the file to be imported (or use the '**Browse...**' button to achieve the same). BLASTGrabber will suggest a name for the to-be-generated BLASTGrabber input file, using the same path and name as the BLAST output file, but using a 'bgr' filename extension. You are free to change this default name, including the default file extension.

Once you have specified the file names, you start importing by clicking the '**Import**' button. If you have checked the '**Open imported file'** checkbox, your file will be opened after the importing is done. Otherwise, you have to open the generated input file ('**File->Open file...**').

During import, you can see the approximate import progress by looking at the progress bar or the currently imported line number (these are both just indirect indications, the actual import might stop slightly before or after reaching 100%).

After importing (and possibly opening) your file, you can register the BLAST output file (and other associated files) in the opening '**Preferences**' window. BLASTGrabber uses these file references if you want to extract alignments or query sequences in the subsequent analysis.

Import-related warnings might have been written to the 'ImportWarnings.txt' file (located in the BLASTGrabber input file folder) after import. You can choose import options and which warnings to report in the '**Import BLAST output file**' tab of the preferences window ('**Edit->Preferences...**').

These warnings can normally be ignored in subsequent analyses (but you might want to store them for latter reference – the 'ImportWarnings.txt' file will be overwritten by subsequent imports).

Errors might be caused by non-supported BLAST formats (such as HTML BLAST output files). Currently, BLASTGrabber supports XML and text BLAST output of all standard BLAST programs (“blastn“,“blastp“,“blastx“,“tblastn“ and “tblastx“). Both the older BLAST version 2.2.24 and the newer BLAST+ output is supported.

Apart from the actual alignments produced by the BLAST algorithm, most of the content of your BLAST output file is included in the BLASTGrabber input file. After selecting sequences of interest, you can view the original BLAST alignment inside BLASTGrabber by browsing to the BLAST output file. It is thus advisable to store the BLAST output file in the same folder as the generated BLASTGrabber input file. (This also includes the FASTA query file used as input for the BLAST search, as this also can be used from BLASTGrabber so as to view the actual query sequences).

Advanced import functionality

Splitting a large BLAST output file

If the memory limitations prevent the import of a large BLAST output file, you can split the file into several smaller files (‘**File->Split file...**’). After selecting the BLAST output file in question and clicking the ‘**Analyse BLAST output file**’, you can specify the number of parts you want in addition to selecting a result files folder:

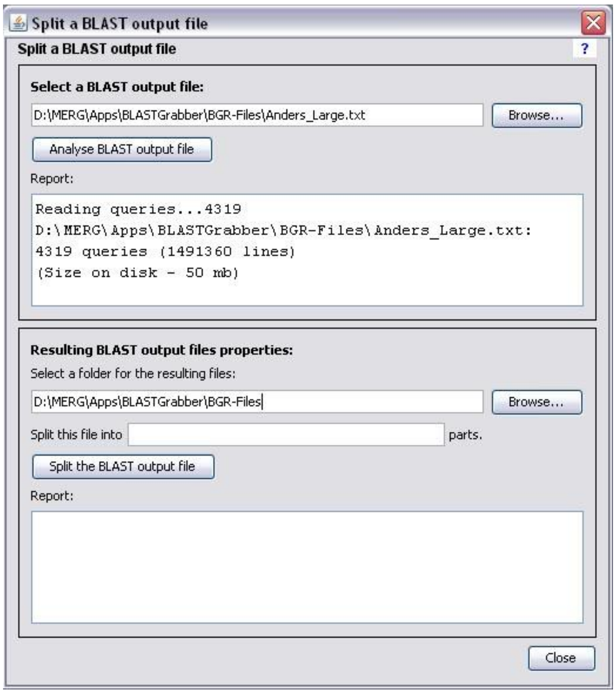

The resulting files will receive the same name as the original BLAST output file followed by part numbers.

The BLAST output file is split on a per-query basis; i.e. each part always starts with a new query (never with sequences belonging to a query from some former part). The number of queries in the different part files need not be identical; a BLAST output file with five queries that is split into three parts would result in two part files with two queries each, and one part file with one query.

NOTE: Importing BLAST output files demands more memory than opening the resulting BLASTGrabber input files. If memory limitations prevent you from importing a large BLAST output file, you can try splitting this file into several parts, importing those parts individually (i.e. creating corresponding BLASTGrabber input files) and finally merging the BLASTGrabber input files into one file corresponding to the original BLAST output file.

NOTE: If merging the imported parts at later on (see below), you can benefit from the ‘**All queries are distinct**’ option since BLAST output files always are split between queries.

Merging several BLASTGrabber input files

It is possible to merge several BLASTGrabber input files, resulting in one input file containing all the BLASTGrabber data. For instance, after copying distinct subsets of your data to different clipboards and saving these as BLASTGrabber input files, you might want to merge these subsets later on in your analysis.

In the '**Merge BLASTGrabber input files**' window ('**File->Merge files...**'), you must add the input files you want to merge, followed by specifying the file name of the resulting merged file:

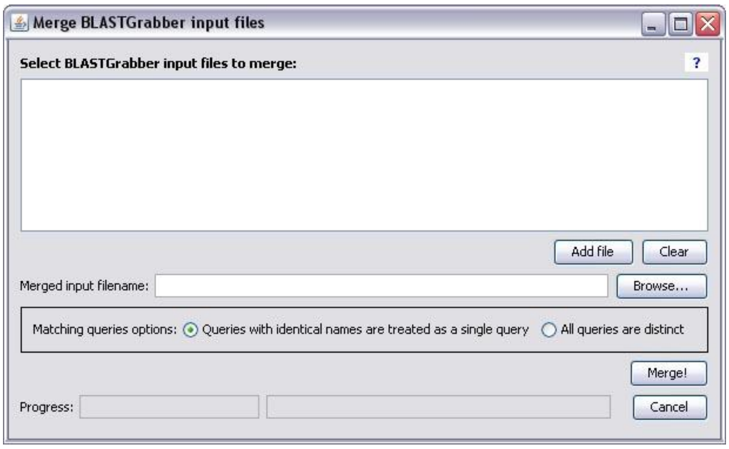

If you are certain your queries are distinct (i.e. every query appears only in one of the to-be-merged files), you can select the '**All queries are distinct**' option. Otherwise, choose the '**Queries with identical names are treated as a single query**' option. In the latter case, if two (or more) to-be-merged files contain queries with identical names, this query will be written to the resulting file only once, together with all associated sequences.

NOTE: Unless you know that queries with identical names actually refer to distinct queries, the '**Queries with identical names are treated as a single query**' option is the safer option - albeit at a performance penalty.

Inferring taxonomy identifiers

BLASTGrabber supports the visualization and selection of BLAST hits based on the NCBI taxonomy. BLASTGrabber can assign taxonomy IDs to BLAST hits by either

- mapping the BLAST hit gi numbers to the tax IDs
- parsing the BLAST hit headers for species names
- or by assigning all hits to one manually selected taxonomy group

After importing your BLAST output file the normal way, selecting '**File->Infer taxonomy ids...**' opens the corresponding window (taxonomy information must be loaded at start-up):

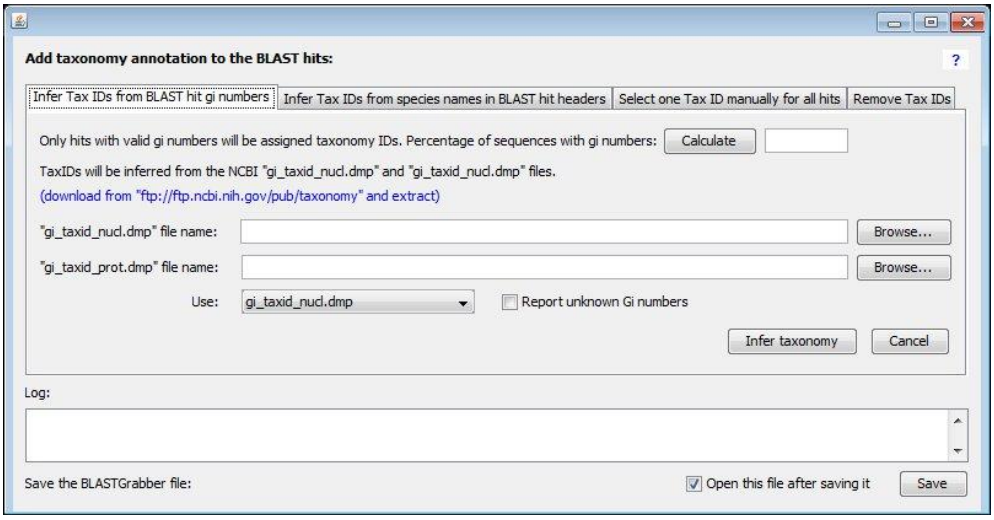

For the first alternative, the two mapping file files must be downloaded from the NCBI ftp site (the URL is given in the program). The file involved in the mapping step is determined automatically based on the type of BLAST program used.

If inferring tax IDs based on parsing the BLAST hit headers, BLASTGrabber is matching words in the header against the (scientific) NCBI taxonomy terms. The longest match will be selected as the relevant tax ID. Often, the species names are given in a standardized way, such as enclosed in square brackets. Optionally, regular expression syntax can be used to extract this specific location.Finally, the user may manually choose a taxonomy group and assign the associated tax ID to all BLAST hits.

Sequences that could not have their taxonomy id identified will be assigned directly to the taxonomy root node. Tax IDs will only be assigned to BLAST hits without such identifiers. Thus, assigning tax IDs may be done in multiple rounds, for instance using the gi numbers in the first step, and subsequently inferring the remaining tax IDs based on the BLAST hit header information.

Click '**Save**' to save the resulting *.bgr file now containing taxonomy ids (leaving the '**Open this file after saving it**' check box will load the resulting file after saving it, closing the currently open BLASTGrabber file).

NOTE: Only hits with valid gi numbers will be assigned tax IDs. If searching standard NCBI databases, gi numbers will be imported if choosing XML as the BLAST output format. The BLAST textual output might not always include gi numbers in the BLAST headers.

A first look at your data

Summary

In order to get a first look at your data, you can open the “Summary” window (‘**View->Summary**):

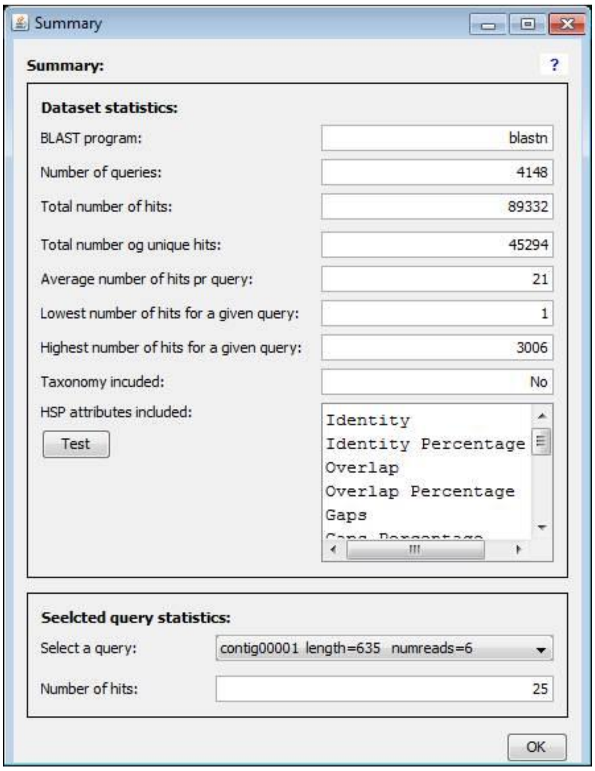

This window gives an overview over your data, including what BLAST program was used, which HSP attributes are imported, and whether taxonomy information is present in your BLAST output data.

‘**Total number of hits**’ represents the sum of the number of hits for each query. In contrast ‘**Total number of unique hits**’ counts each hit only once. Thus, these two numbers differ if a given hit is included for two or more queries.

Also, you can see the number of hit for each your queries by selecting the query of interest in the ‘**Select a query**’ combo box.

Taxonomy

The taxonomy window (‘**View->Taxonomy**’) displays a summary of taxonomy information, if present in your BLAST output data (and if you have allowed BLASTGrabber to load taxonomy).You can see the number of hits for all taxa present in your data, in addition to seeing all hit as located in the NCBI taxonomy tree:

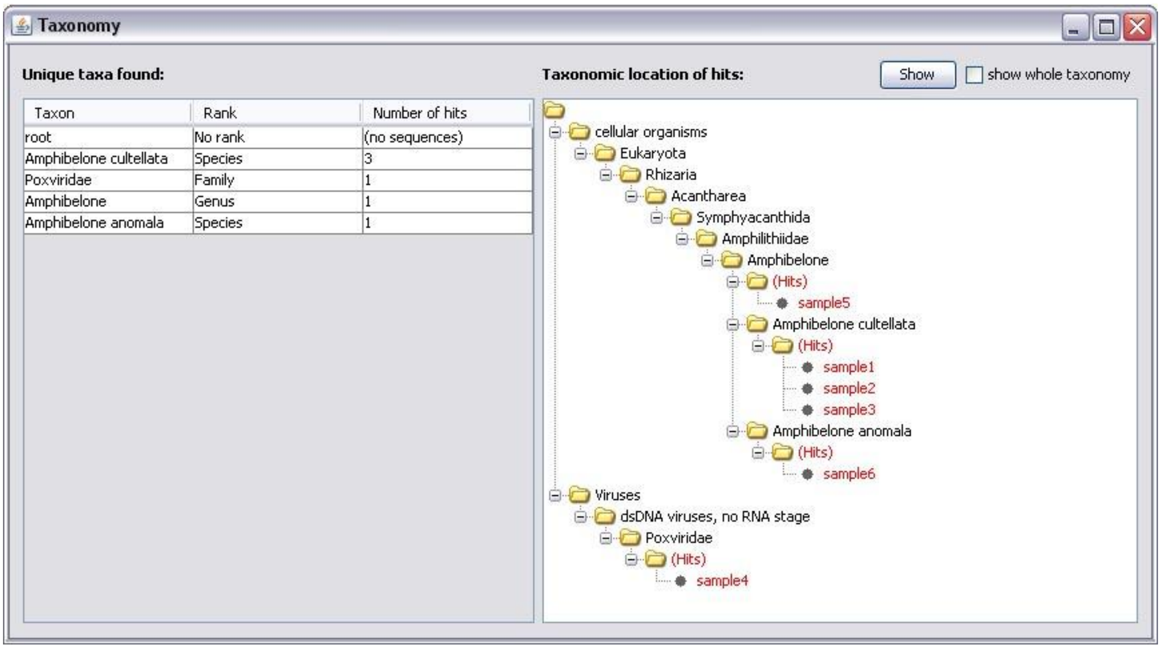

(The ‘**show whole taxonomy**’ checkbox controls whether the whole taxonomy is displayed, or only the taxonomy subset actually of interest in relation to your data).

Analysing your data in the sequence viewer

You can see what regions of your queries have accumulated hits by opening the sequence viewer window (‘**Sequence selection->Sequence viewer**’). After selecting a data source (such as the entire dataset), this window displays a list over all your sequences, including the number of hits for each of them:

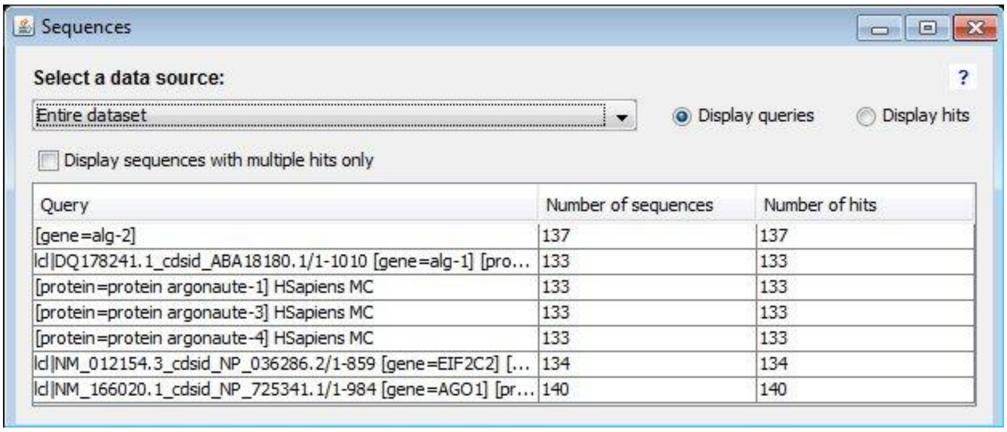

By clicking on one of them, you open the actual analysis window, displaying the distribution of hits along the query sequence. The query sequence (including character numeration) is displayed on the first line; the other lines display hits coloured in yellow:

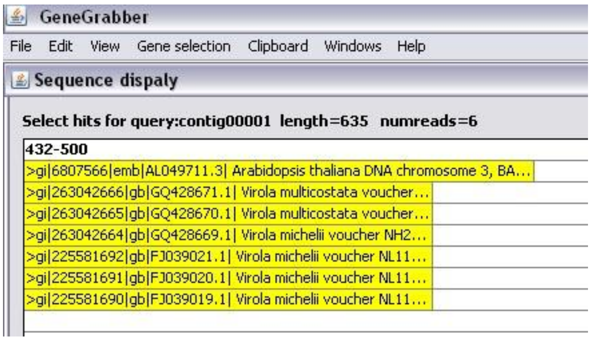

For long sequences, you must enter the precise interval along the sequence which you want to inspect:

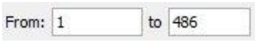

NOTE: intervals always will be cropped so as to start and end with a hit.

You can set a desired numeration interval with the ‘**Guide interval width**’ textbox, and zoom in or out using the ‘**Zoom**’ spinner (large changes in zoom level can be entered directly into the zoom textbox; move away from the textbox for instance by pressing the “Tab” key in order to apply the change):

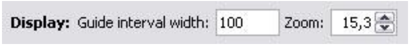

What actually is displayed in the hits is selected by the ‘**Display**’ combo box – “FASTA header” was selected in the example above. You can select HSP attributes such as e-values or identities in order to assess the relevance of the hit:

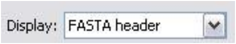

In order to further facilitate the identification of interesting sequences, you can apply heat map rendering by right-clicking in the display area and selecting '**Toggle Heatmap**':

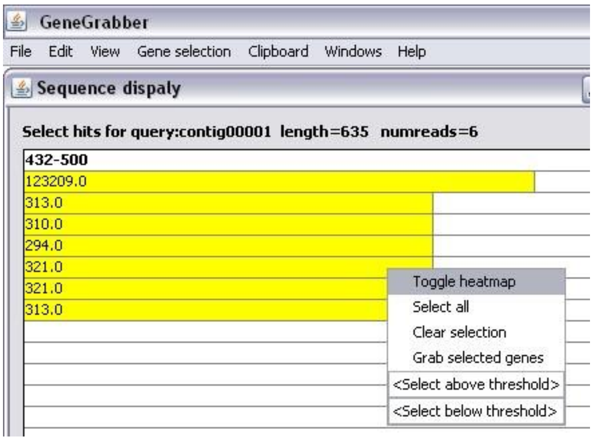

The heat map will assign colours to your hits, dependent upon the HSP attribute selected and the minimum and maximum of displayed data. The '**Heatmap legend**' box in the lower right corner displays the legends for the colours used: in the above example, the values range from a minimum of 87.5 (blue) to a maximum of 113 (white):

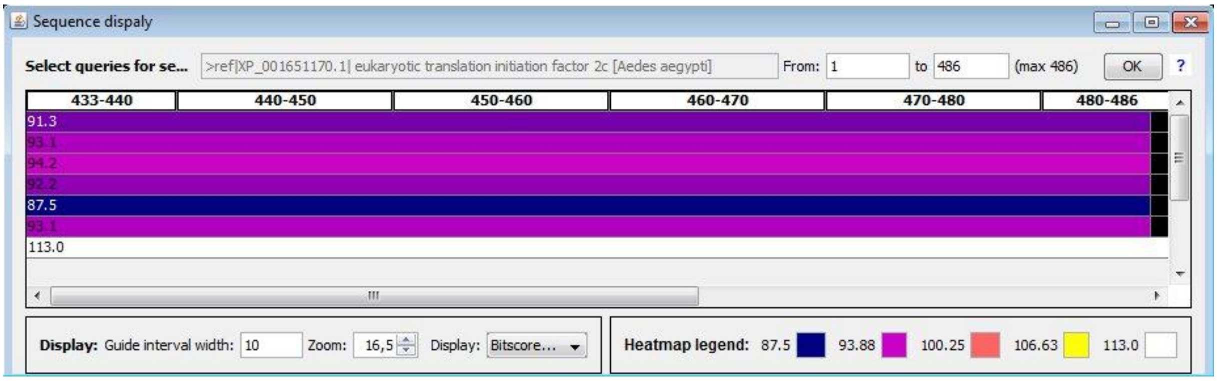

You can select sequences of interest by clicking on them. Also, by accessing the right-click menu you can select or de-select all sequences, in addition to selecting sequences above or below a certain threshold value. For instance, you might want to select all sequences under a certain E-value threshold, or above a certain identity level. By right-clicking the selected sequences and choosing '**Grab selected sequences**', you can copy your sequences to the clipboard, so as to process them further (see below):

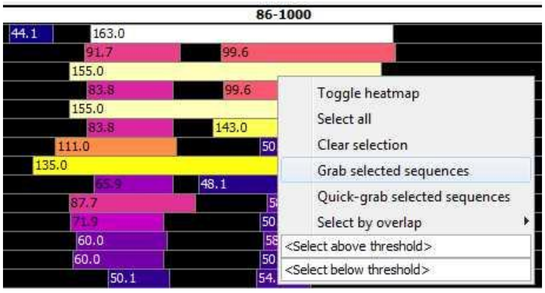

The above examples show how hit are mapped to the sequence of a query. By selecting the '**Display Hits**' radio button (rather than the default '**Display queries**' radio button) in the sequence viewer list, you can visualize your sequences the other way around:

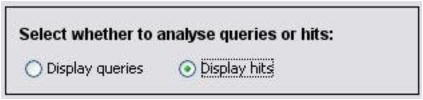

This displays a list over all hits rather than queries, and clicking an entry in the list displays how your queries map to the specific hit in the same manner as explained above.

Analysing your data in the matrix viewer

Setting up the matrix

In order to arrange your BLAST hits according to some attributes such as E-value, identity or length, you can use the matrix viewer (‘**Sequence selection-> Matrix viewer**’). Data will be arranged in a 2-dimensional matrix; you have to define which HSP attributes to use, and the size of the resulting intervals:

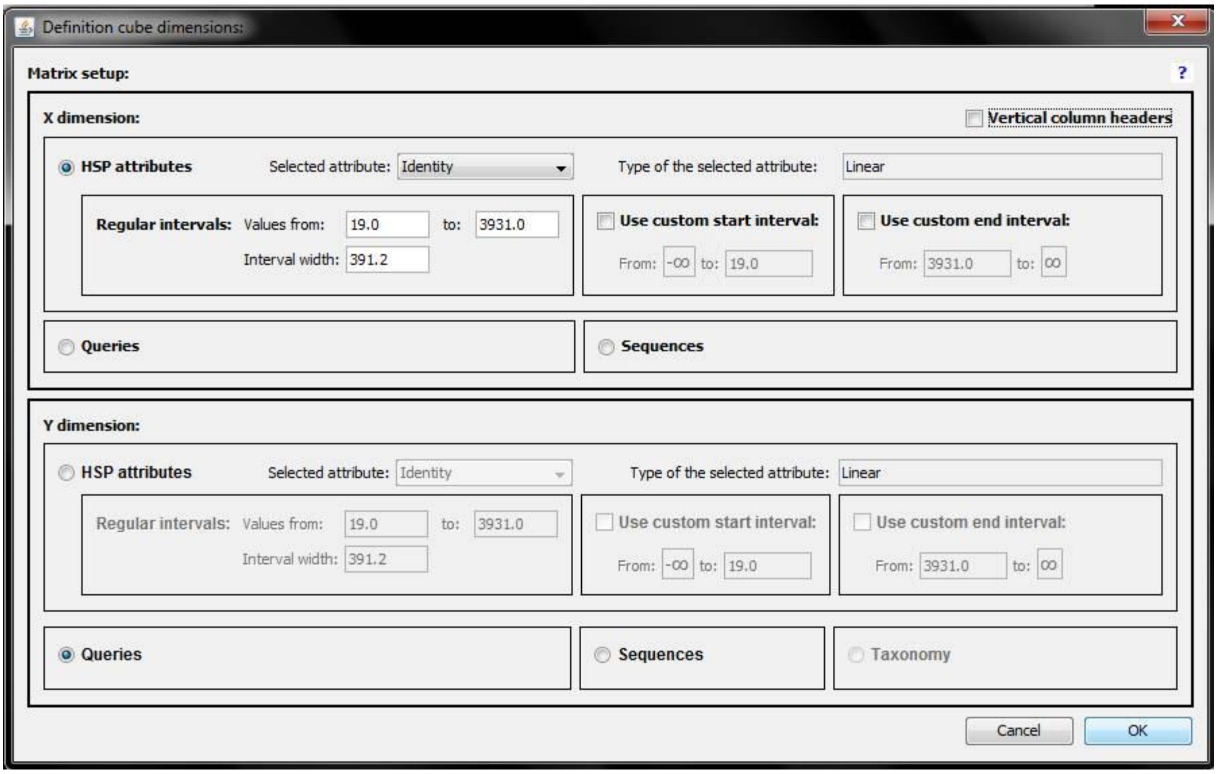

You can select HSP attributes both as the X- and as the Y-axis. Once you select a given attribute in the ‘**Selected attribute**’ combo box, the minimum and maximum values present in your data are automatically entered into the ‘**Values from**’ and ‘**to**’ text boxes; the interval width is set to a value corresponding to 10 intervals.

You can also use custom start and end intervals. For example, you might specify ‘**Values from**’ and ‘**to**’ as 19 and 1000, respectively (and also setting ‘**Interval Width**’ value to a suitable number). Selecting the ‘**Use custom end interval**’ checkbox means that all hits with identities higher than 1000 are grouped together (the custom end interval ‘**from**’ value is automatically set to 1000, but can be changed manually):

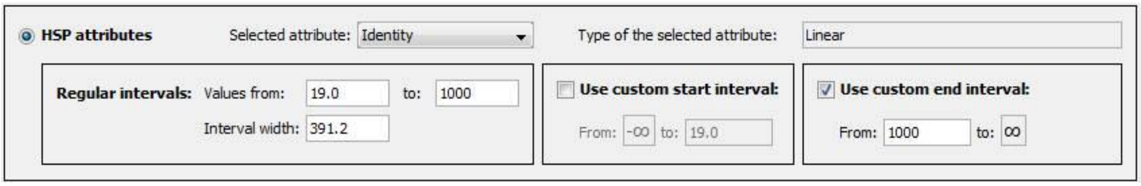

The default setup for the matrix uses ‘**Queries**’ as the Y axis. Thus, if you have 100 queries represented in your BLAST output file, you will see the 100 queries represented under each other.

Instead of using ‘**Queries**’ (or an attributes) for the Y axis, you can also select the ‘**Sequences**’ radio button. However, this is advisable only for rather small result sets – moderate to big BLAST output files can contain many thousand unique hit sequences, creating a matrix too big for both human and computer processing.

If your BLAST output data contains taxonomy Ids (and if you have allowed BLASTGrabber to load taxonomy information), you can order your hits according to taxonomy.

(Taxonomy Ids are only included in your data if you have used the “bioportal.uio.no” BLAST version)

Matrix visualization and data selection

After selecting suitable X- and Y-axis definitions, you will see the matrix viewer. This viewer supports much of the same functionality as the sequence viewer (see above), such as heat map rendering and data selection. In the following example, BLAST hits are ordered according to their E-values along the X axis, and according to their identity percentage along the Y-axis:

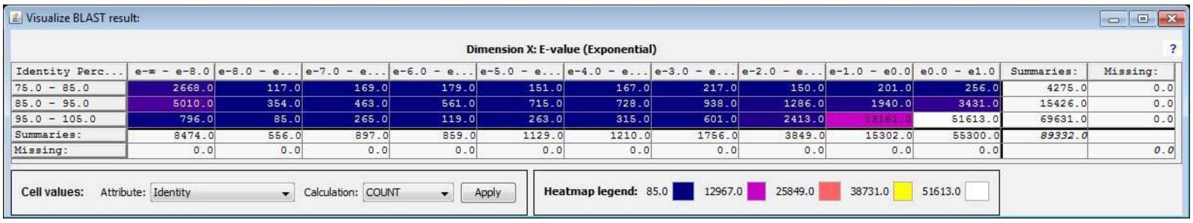

Identity Perc...	e-∞ - e-8.0	e-8.0 - e...	e-7.0 - e...	e-6.0 - e...	e-5.0 - e...	e-4.0 - e...	e-3.0 - e...	e-2.0 - e...	e-1.0 - e0.0	e0.0 - e1.0	Summaries:	Missing:
75.0 - 85.0	2668.0	117.0	169.0	179.0	151.0	167.0	217.0	150.0	201.0	256.0	4275.0	0.0
85.0 - 95.0	5010.0	354.0	463.0	561.0	715.0	728.0	938.0	1286.0	1940.0	3431.0	15426.0	0.0
95.0 - 105.0	796.0	85.0	265.0	119.0	263.0	315.0	601.0	2413.0	13161.0	51613.0	69631.0	0.0
Summaries:	8474.0	556.0	897.0	859.0	1129.0	1210.0	1756.0	3849.0	15302.0	55300.0	*89332.0*	
Missing:	0.0	0.0	0.0	0.0	0.0	0.0	0.0	0.0	0.0	0.0		*0.0*

The numbers appearing in the matrix cells are controlled by the '**Fact dimension**' settings; the example above uses the '**COUNT**' as '**Fact calculation**'. This means that for each cell, the numbers of hits are displayed. For instance, 51613 hits have E-values between 1 and 10, while also having an identity percentage higher than 95% (the lower right cell with white background colour).

A number of fact dimension calculations are available, such as the average ('**AVG**'), sum ('**SUM**') or variance ('**VAR**'). The calculation is performed upon the HSP attribute selected in the '**Attribute'** combo box (note that the selected attribute does not matter if selecting '**COUNT**' as the calculation mode):

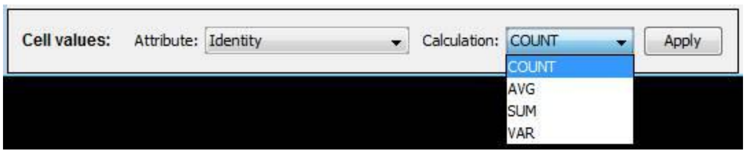

In this way, you can analyze your data using three dimensions at once, for instance by selecting E-value and identity percentage as the X- and Y-axis (as used above), and a third dimension to be displayed in the cells themselves:

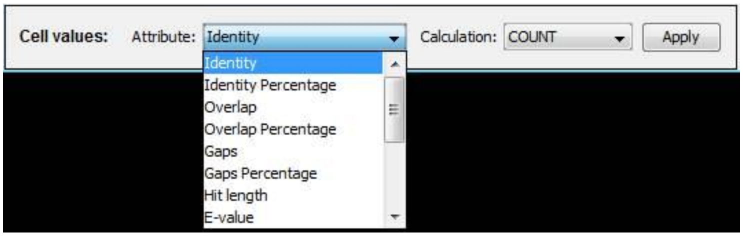

You can order vertical order of the matrix rows by clicking on a column header. Also, by right-clicking on a column header you will select the whole column, possibly facilitating the selection of all significant hits (if E-values are used as the X-axis attribute).

NOTE: The E-value attribute behaves somewhat different from other the HSP attributes available, due to its exponential nature. As is visible in the example above, E-values are arranged according to their exponent. If your data contains E-values between 10 (1e1) and 0.001 (1e-3), you should set up the dimension with a maximum of 1 and a minimum of -3. The calculation results displayed in the matrix cells, however, are always the actual values.

Analysing sequence descriptions with the description viewer

Using the description viewer

You can use the description viewer ('**Sequence selection-> Description viewer**') in order to understand more about what selected sequences are, where they come from or which organism they belong to. Alternatively, you can also select queries based on query names.

After selecting a data source (the entire dataset loaded in BLASTGrabber or data copied to a clipboard), the description viewer optionally breaks down all the FASTA header descriptions (such as "`>gi|300795987|ref|NP_001178694.1| protein argonaute-1 [Rattus norvegicus]`") into single words. This is controlled by the '**word separator expression**' text box (click the '**Display settings**' button to make it visible). The default '' (blank space) causes the entire headers (or query names) to be displayed. The number of times a specific word is found (its occurence) in the selected data source is presented along with its relative percentage:

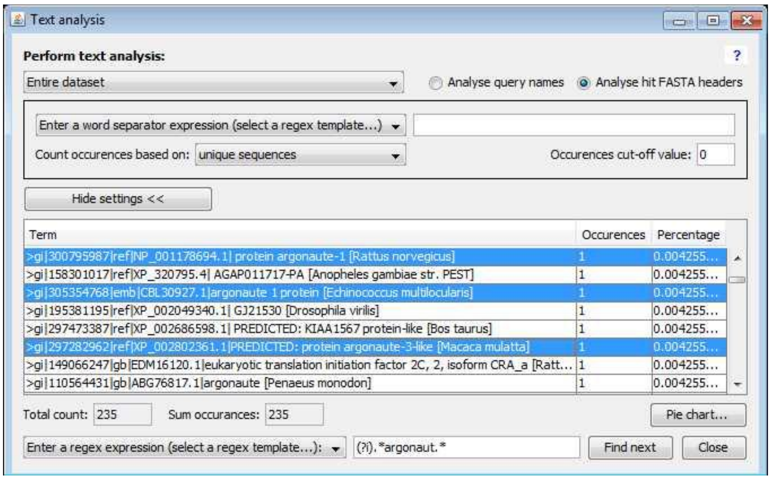

The user can choose between counting the words in each BLAST hit sequence only once ('unique sequences') or each time that sequence is hit by a query ('all sequences'). Thus, presuming a data source of only two queries that hit the same sequence, the description words in this sequence will receive an occurrence count of "1" in the first case versus an occurrence count of "2" in the second.

It is possible to search (and select) words of interest by entering a regular expression into the "**Enter a regex expression**" text box (the user can select regular expression templates from the dropdown list, making it easy to obtain the correct syntax). Pressing the '**Find next**' button scrolls down the list to the next matching item.

NOTE: BLASTGrabber is a Java-based program. As such it uses Java-style regular expression which might differ from other dialects such as implemented in Perl or other languages.

After selecting words of interest (either by manually clicking the corresponding table row, or entering a regex search expression), you can copy the matching sequences to a clipboard by right-clicking the table and selecting "Grab selected sequences". If your data source is a clipboard, you cannot copy to the same clipboard (you already have the selected sequences on this clipboard). In this case you should add another clipboard in the '**Clipboard->Clipboard administration**' window (see further down).

Configuring the description viewer settings

In the '**Edit->Preferences**' window, '**Description viewer**' tab, the user can add words to be ignored in the description viewer window, such as general words like 'and' or 'a'.

Working with clipboards

Displaying selected sequences

Using the sequence viewer, the matrix viewer or the description viewer, you can select your sequences of interest and "grab" them by right-clicking and selecting "**Grab selected sequences**". This causes the sequences to be copied to the BLASTGrabber-specific clipboard. You can view the contents of your clipboard by clicking '**Clipboard-><Default>**':

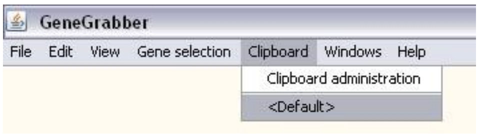

(Selecting "**Quick-grab selected sequences**" will delete all reset the default clipboard, copy the selected sequences to it and immediately display this clipboard.)

It is possible to define more than one clipboard in BLASTGrabber (see below). In that case, you will see the names of your additional clipboards listed underneath the default clipboard.

After clicking the '**Display queries**' button, the clipboard window displays the selected sequences order under their respective queries. By expanding a sequence, the HSP attributes for that hit are displayed:

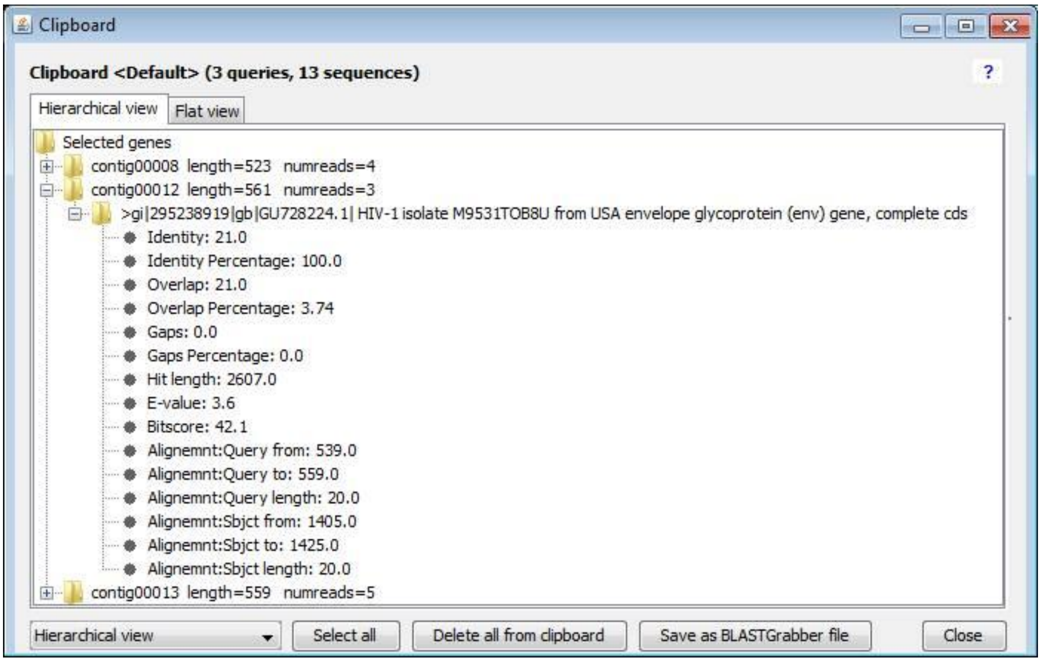

The clipboard content can be saved by clicking the '**Save as BLASTGrabber file**' button. This creates a file that can be opened with the BLASTGrabber program.

Selecting another option than '**Display sequences**' in the combo box shows a text-based list over all selected sequences (gi-numbers, accession numbers or the total FASTA header can be selected for display). This display mode facilitates the copy-and-paste export of the selected sequences.

The selected sequences can also be directly downloaded from the NCBI website by selecting, right-clicking and choosing '**Download selected sequences from NCBI**'. Here, the user must choose a

target folder for the download; the NCBI format of the download (FASTA format, genbank or other) is determined in the '**Edit->Preferences**' window.

The user can also choose to view the relevant alignment in the original BLAST output file. Initially, the user must give the BLAST output filename; this filename is remembered for subsequent requests. A similar function is available for the queries; after giving the FASTA file used as the query input file for the BLAST search, the corresponding query sequence is displayed:

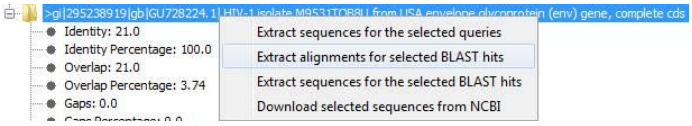

Clipboard administration

In addition to the default clipboard, you can create additional clipboards using the '**Clipboard->Clipboard administration**' window:

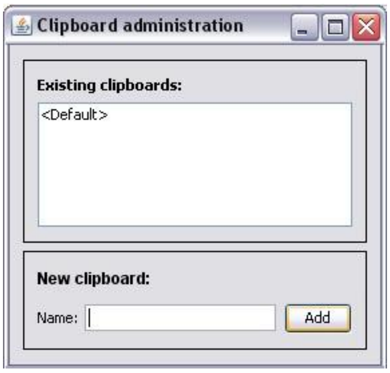

If multiple clipboards have been created, after grabbing sequences you will be presented with a choice of which clipboard to use.

NOTE: clipboard setup and contents are not preserved across BLASTGrabber sessions. Thus, if you open another BLASTGrabber input file (or quit the BLASTGrabber program), the setup and contents of your clipboards are deleted. Make sure to save clipboard content if you do not want to lose your selection.

Editing your preferences

The preferences window ('Edit->Preferences') allows the configuration of BLASTGrabber tools. It contains four tabs:

- **Import BLAST output file**: settings used when importing a BLAST output file.
  - The '**Infer identical BLAST hit...**' options define whether a given sequence is treated as one single sequence or two distinct sequences if hit by two (or more) queries. The default setting is '**Entire FASTA header**', meaning that if two queries contain hits with identical FASTA headers, these two sequences will be assumed to be identical and one single sequence. The default setting of '**Entire FASTA header**' (and '**Infer identical BLAST hit...**' set to 'checked') should only be changed by experienced users.
  - The '**E-value'** option determines how BLAST hits with '0.0' as e-values are imported into BLASTGrabber. (When selecting 'E-value' as the HSP attribute in the matrix viewer, matrix setup depends on the e-value exponent for cell assignment, thus an exponent reflecting a very small value is needed for zero e-values.)
  - The '**Import warnings**' options determine what gets written to the import warnings file. For instance, it might be desirable to record which queries did not have any associated hits, especially if not importing these queries.
  - The '**Missing hits**' option determines whether queries with no hits are imported into BLASTGrabber.
- **Download sequences**: options for the download of sequences.
  - The '**Select download mode**' determines the format of the downloaded file
  - The '**NCBI nucleotide db**' and '**NCBI protein db**' strings determine which NCBI database is used when downloading nucleotide and protein sequences, respectively. The default setting should only be changed by experienced users.
- **File references**: BLASTGrabber can associate files such as BLAST output files, query files or custom FASTA database files with a particular *.bgr file, thereby allowing the access to information not included in the *.bgr file. Examples thereof include BLAST alignments or query sequences.
  - The displayed file names relate to the currently loaded *.bgr file, and can be changed at will.
  - File name references not associated with the currently loaded *.bgr file can be deleted by pressing the '**Delete**' button. This should be done if stored reference file names exceed the storage capacity of the preferences cache (an error message to this effect will be given).
- **Description viewer**: configuration of the description viewer window.
  - This tab allows the user to filter and control how the text analysis of the FASTA header descriptions is performed. See also 'Analysing sequence descriptions with the description viewer'.

Appendix

BLASTGrabber plugin architecture

BLASTGrabber supports third-party plugin design by defining two interfaces used for communication to and from BLASTGrabber. At start-up, the program will load all Java JAR file plugins that are found in the ‘plugins’ subfolder. Plugins can be invoked from BLASTGrabber upon user actions; either by clicking the ‘plugin’ menu and selecting a plugin name, or by right-clicking the selected hits in the clicpboard and subsequently selecting a relevant plugin. The plugin can communicate with the BLASTGrabber program by accessing the reference given in the ‘initialize’ function.

The BLASTGrabber.Facade interface definition:

```
package BLASTGrabber;
import java.util.ArrayList;
import java.util.HashMap;
import java.util.Hashtable;
import javax.swing.JDesktopPane;
import javax.swing.JInternalFrame;

public interface Facade {
    public JDesktopPane getDesktopPane();
    public ArrayList<String> getBLASTAlignments(HashMap<String, BLASTGrabberQuery> queries);
    public ArrayList<String> getFASTAQueries(HashMap<String, BLASTGrabberQuery> queries);
    public ArrayList<String> getFASTACustomDBSequences(HashMap<String, BLASTGrabberQuery>
queries);

    public class BLASTGrabberQuery{
        public int BLASTGrabberID=-1;
        public String Name="";
        public ArrayList<BLASTGrabberHit> Hits=new ArrayList<BLASTGrabberHit>();
    }

    public class BLASTGrabberHit{
        public int BLASTGrabberID=-1;
        public String SequenceHeader;
        public ArrayList<BLASTGrabberStatistic> Statistics= new
ArrayList<BLASTGrabberStatistic>();
    }

    public class BLASTGrabberStatistic{
        public String Name="";
        public String Key="";
        public double Value=0;
    }

}
```

The Plugin.Facade interface definition:

```
package Plugin;
import BLASTGrabber.Facade.BLASTGrabberQuery;
import java.util.HashMap;

public interface Facade {
    public void initialize(BLASTGrabber.Facade facade);
    public String getName();
    public void displayMain();
    public boolean doesProcessSelectedClipboardData();
    public void processSelectedClipboardItems(HashMap<String, BLASTGrabberQuery> queries);

}
```
